# Computed Tomography-Guided Core Needle Biopsy of Cardiac Angiosarcoma

**DOI:** 10.5935/abc.20180064

**Published:** 2018-05

**Authors:** Luis Gorospe, Alberto Cabañero-Sánchez, Gemma María Muñoz-Molina, Ana María Ayala-Carbonero, María Ángeles Fernández-Méndez

**Affiliations:** Ramón y Cajal University Hospital, Madrid - Spain

**Keywords:** Hemangiosarcoma/diagnosis, Hemangiosarcoma/pathology, Biopsy, Large-Core Needle, Tomography, X-Ray Computed, Neoplasm Staging

**Figure 1:**
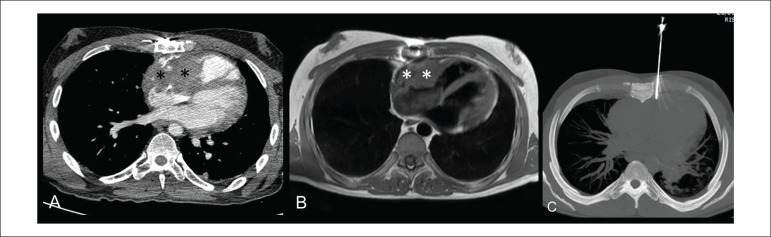
A) Axial contrast-enhanced CT image showing heterogeneous mass (asterisks) infiltrating the right atrium, the right atrioventricular groove, and the right ventricle; B) Axial T1-weighted MR cardiac image showing mass (asterisks) infiltrating the right cardiac chambers; C) Axial unenhanced CT MIP (maximum intensity projection) image showing core-needle biopsy, with the tip entering the cardiac mass.

A 34-year-old man was referred to our institution after an echocardiography performed at another center because of tachycardia (atrial flutter), which showed heterogeneous pericardial mass infiltrating the right chambers. Thoracic computed tomography (CT) and cardiac magnetic resonance (MR) imaging were performed for more accurate assessment of the exact tumor location, size, and potential infiltration of other cardiac and mediastinal structures. CT (Figure 1A) and MR imaging (Figure 1B) confirmed an 8-cm ill-defined heterogeneous enhancing pericardial mass infiltrating the anterior and superior walls of the right atrium and extending to lateral and inferior walls of the right ventricle, consistent with cardiac angiosarcoma. The patient was deemed inoperable, as the mass also invaded the superior vena cava, aortic root, and epicardial fat. CT-guided core needle biopsy of the cardiac mass was the method of choice for histological verification of tentative diagnosis. Once the patient signed the informed consent and was put in supine position, an 18-gauge biopsy needle was driven between the left thoracic internal arteries and the left border of the sternal body (Figure 1C) and a tissue specimen was safely obtained from the beating heart without adverse events. The procedure was performed by an experienced interventional thoracic radiologist under local anesthesia and in the presence of a thoracic surgeon. CT images obtained immediately after biopsy showed no post-procedural complications. Preliminary histological analysis performed on-site by a pathologist determined the adequacy of the tissue specimen. Final histopathologic diagnosis was high-grade cardiac angiosarcoma. To the best of our knowledge, only one case of a CT-guided core needle biopsy of a cardiac angiosarcoma involving the right chambers has been previously reported in English-language scientific literature.

